# Exploring the Role of Ketamine Sedation in Critically Ill Patients: A Comprehensive Review

**DOI:** 10.7759/cureus.65836

**Published:** 2024-07-31

**Authors:** Souvik Banik, Sheetal Madavi

**Affiliations:** 1 Anesthesiology, Jawaharlal Nehru Medical College, Datta Meghe Institute of Higher Education and Research, Wardha, IND

**Keywords:** hemodynamic stability, respiratory function, pharmacodynamics, critical care, sedation, ketamine

## Abstract

Sedation management in critically ill patients is a critical component of intensive care, aiming to balance the need for comfort and immobilization with preserving vital physiological functions. Ketamine, known for its dissociative anesthetic properties, has emerged as a promising alternative to traditional sedatives due to its unique pharmacological profile. This review explores the pharmacodynamics, clinical applications, benefits, challenges, and current evidence surrounding ketamine as a sedative agent in intensive care settings. Key advantages of ketamine include its ability to maintain respiratory drive and hemodynamic stability, making it particularly suitable for patients requiring continuous monitoring and intervention. The review discusses its role in sedation protocols, compares its effectiveness with other sedatives, and highlights potential areas for further research and optimization. By elucidating the complexities and advancements in ketamine sedation, this review aims to inform clinical practice and contribute to improved outcomes for critically ill patients.

## Introduction and background

In critical care settings, the effective management of sedation is crucial for ensuring patient comfort, facilitating necessary medical procedures, and minimizing complications associated with prolonged immobility and discomfort [1]. The choice of sedative agents plays a pivotal role in achieving these goals, as they must balance the need for adequate sedation with the preservation of respiratory function and hemodynamic stability [2].

Ketamine, developed initially as a dissociative anesthetic, has garnered significant interest in recent years for its unique pharmacological properties that make it suitable for sedation in critically ill patients [3]. Unlike traditional sedatives such as benzodiazepines and propofol, which may depress respiratory drive and cardiovascular function, ketamine offers a distinct advantage by preserving these vital parameters while providing effective sedation. This characteristic is particularly advantageous in intensive care units (ICUs), where maintaining optimal physiological stability is paramount to patient outcomes [4].

The purpose of this review is to comprehensively explore the role of ketamine as a sedative agent, specifically in critically ill patients. By delving into its pharmacodynamics, clinical applications, benefits, challenges, and current evidence base, this review aims to provide a nuanced understanding of ketamine's utility in sedation strategies within the intensive care environment. Furthermore, it seeks to identify areas of future research and development to enhance the integration of ketamine into standard sedation protocols, ultimately improving patient care and outcomes in critical care settings.

## Review

Pharmacology of ketamine

Mechanism of Action

The primary mechanism of action of ketamine is its role as a non-competitive antagonist of the N-methyl-D-aspartate (NMDA) receptor. Ketamine binds to the phencyclidine (PCP) site within the NMDA receptor channel, decreasing channel opening time and reducing the amplification of repeated stimuli ("wind-up") that can contribute to pain perception. This NMDA receptor antagonism underlies ketamine's anesthetic, analgesic, and antidepressant effects [5]. Besides NMDA receptor antagonism, ketamine interacts with opioid receptors, monoaminergic receptors, muscarinic receptors, and voltage-sensitive calcium channels. Unlike other general anesthetics, ketamine does not interact with gamma-aminobutyric acid (GABA) receptors. Ketamine enhances descending serotonergic inhibitory pathways, contributing to analgesic effects [6]. Ketamine can produce cardiovascular stimulation, bronchodilation, and occasionally transient respiratory depression. This unique pharmacological profile, preserving respiratory and cardiovascular function, makes ketamine a valuable anesthetic agent in critical care settings [7].

Pharmacokinetics and Metabolism

Ketamine has a rapid onset of action when administered intravenously, reaching peak plasma concentrations quickly. The intramuscular route provides high bioavailability, approximately 93%, with peak levels occurring within 5-30 minutes. Oral bioavailability is lower, ranging from 16% to 29%, due to extensive first-pass hepatic metabolism. Intranasal and rectal administration offer better bioavailability at 45%-50% and 25%-30%, respectively. Ketamine is highly lipophilic and has a large volume distribution of 2-3 L/kg [5]. Ketamine is primarily metabolized by the CYP3A4 enzyme, with secondary metabolism by CYP2B6 and CYP2C9. The main metabolic pathway involves N-demethylation to norketamine, an active metabolite. Norketamine is further metabolized to hydroxynorketamine and other minor metabolites. Metabolism occurs in the liver and other tissues, including the kidneys, intestines, and lungs. The S(+) enantiomer of ketamine is metabolized more rapidly than the R(-) enantiomer [8]. Ketamine has an elimination half-life of two to three hours and high clearance at 12-20 mL/min/kg, approaching liver blood flow. Most ketamine and its metabolites are excreted in the urine, with 91% of administered radioactivity appearing over five days. Only about 20% is excreted as the parent drug or major metabolites. Recent studies suggest that some of ketamine's metabolites, such as hydroxynorketamine, may also contribute to its rapid antidepressant effects. The pharmacokinetics and metabolism of ketamine are complex, involving rapid absorption, extensive distribution, high clearance, and active metabolites that may have therapeutic relevance [5].

Unique Properties Relevant to Critical Care Settings

Ketamine's unique pharmacological profile makes it an invaluable tool in critical care settings. Unlike other sedatives such as benzodiazepines and propofol, ketamine does not significantly depress respiratory drive; in fact, it can cause bronchodilation, making it particularly useful for patients with respiratory conditions like asthma. Additionally, ketamine stimulates the cardiovascular system, increasing heart rate and blood pressure, which helps maintain hemodynamic stability in critically ill patients [7]. Ketamine provides both analgesia and dissociative sedation, making it suitable for pain management and procedural sedation in the ICU. The dissociative state induced by ketamine is an "all-or-nothing" phenomenon, unlike the continuum of sedation seen with other agents, allowing for more precise titration of the desired sedation level. Some studies suggest that ketamine may also have neuroprotective properties, which could be beneficial in neurological critical care situations, such as traumatic brain injury or status epilepticus [9]. Furthermore, ketamine has been shown to reduce opioid requirements and mitigate opioid-induced hyperalgesia in surgical and trauma patients requiring mechanical ventilation. This opioid-sparing effect is particularly advantageous in the critical care setting, where minimizing opioid use is often a priority [10].

Clinical applications of ketamine in critical care

Sedative Properties and Depth of Sedation

Ketamine is a dissociative anesthetic that induces a trance-like state known as "dissociative anesthesia." At anesthetic doses, ketamine provides profound sedation, pain relief, and amnesia while preserving respiratory function and cardiovascular stability. This unique profile makes ketamine a valuable sedative agent for critically ill patients, as it avoids the respiratory depression and hemodynamic instability associated with other common sedatives like benzodiazepines and propofol [11]. The depth of sedation with ketamine is dose-dependent. At low doses (0.1-0.5 mg/kg), ketamine provides analgesia and mild sedation without inducing a dissociative state. At higher doses (>1 mg/kg), ketamine can induce a dissociative state where patients appear awake but are unaware of their surroundings. This dissociative state is characterized by preserved respiratory function, airway reflexes, and cardiovascular stimulation [4]. Ketamine's ability to maintain respiratory and cardiovascular function makes it particularly well-suited for sedating critically ill patients who require mechanical ventilation, as it avoids the respiratory depression seen with other sedatives [12].

Analgesic Effects and Pain Management in Critically Ill Patients

The available evidence suggests that several non-opioid analgesic agents can be effective adjuncts to opioid-based pain management in critically ill patients. Studies have found that acetaminophen can reduce opioid consumption by an average of 36 mg/day. Low-dose ketamine infusions have been shown to reduce opioid requirements by an average of 37 mg/day. Nonsteroidal anti-inflammatory drugs (NSAIDs) can reduce opioid use by an average of 11 mg/day, though concerns about adverse effects like bleeding and renal dysfunction may limit their use. Other adjuvants, such as carbamazepine, dexmedetomidine, nefopam, and tramadol, have also demonstrated opioid-sparing effects in critically ill patients [13]. While opioids remain the mainstay of pain management in critically ill patients, their use should be optimized. Continuous opioid infusions should be avoided when possible, as they increase the risk of opioid-related adverse effects. Patient-controlled analgesia (PCA) with opioids may allow for more titrated dosing and reduced overall opioid consumption. Careful monitoring for opioid-induced adverse effects like respiratory depression, constipation, and delirium is crucial [14]. Using a combination of opioid and non-opioid analgesics (multimodal analgesia) can provide effective pain control while minimizing opioid-related side effects. This approach aligns with current guidelines, which recommend the use of adjuvant medications in addition to opioids for pain management in critically ill patients [15].

Role in Procedural Sedation

**Figure 1:**
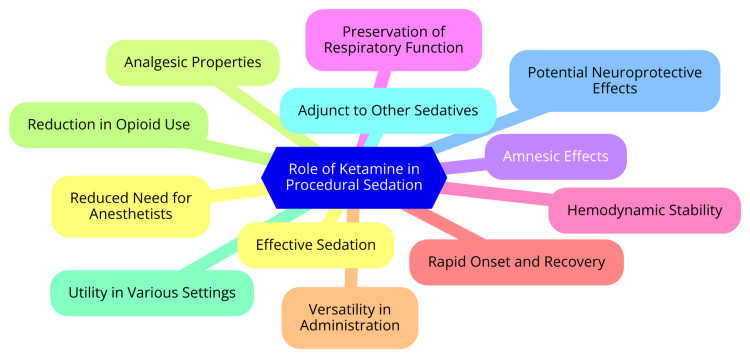
The role of ketamine in procedural sedation Image Credit: Dr Souvik Banik

Ketamine is a widely used agent for procedural sedation, particularly in emergency department settings, offering several key advantages. It is a potent sedative, amnestic, and analgesic agent, providing adequate sedation for short, painful procedures requiring immobilization. Ketamine is effective for procedural sedation in children and adults, with a high success rate in achieving adequate sedation and completing the intended procedure [16]. Unlike many other sedatives, ketamine maintains hemodynamic stability and respiratory function. This makes it particularly useful in critically ill patients or those with cardiovascular instability who may not tolerate the hypotensive effects of other sedatives. Ketamine can be administered intravenously or intramuscularly, with the IV route providing a slightly faster onset and recovery. It does not require IV access and can be given as a single dose or repeated as needed to maintain sedation [7]. The most common adverse effects of ketamine include clonic movements, hypersalivation, laryngospasm, recovery agitation, and vomiting. However, these are generally manageable with appropriate dosing and monitoring, and serious adverse events are rare. Ketamine is an effective, safe, and easy-to-use agent for procedural sedation in emergency departments and other acute care settings. Its unique properties make it particularly useful in respiratory or hemodynamically compromised patients. However, appropriate patient selection, dosing, and monitoring are essential to minimize adverse effects [17]. The role of ketamine in procedural sedation is shown in Figure 1.

Benefits of ketamine sedation in critically ill patients

Preservation of Respiratory Function

Ketamine has been shown to preserve respiratory function and maintain airway patency, making it a beneficial agent for critically ill patients who require mechanical ventilation. Ketamine is a respiratory stimulant that increases genioglossus activity, flow rate, respiratory rate, and duty cycle (adequate inspiratory time). These effects help stabilize airway patency during sedation and anesthesia, reducing the risk of airway collapse. However, the effect of ketamine on upper-airway patency in humans is still poorly understood [18]. Ketamine has a favorable hemodynamic profile, which is crucial for patients with cardiovascular instability. This stability helps maintain respiratory function by ensuring adequate blood flow to the lungs and other vital organs. Ketamine provides effective analgesia and sedation, essential for managing pain and anxiety in critically ill patients, thereby reducing the need for opioids and other sedatives that can depress respiratory drive [7]. Ketamine is used in various clinical settings, including procedural sedation in the emergency department and continuous sedation in the ICU. Its ability to maintain respiratory function makes it a valuable agent for managing critically ill patients who require mechanical ventilation. Overall, ketamine's respiratory stimulant properties, hemodynamically stable profile, and analgesic/sedative effects contribute to its ability to preserve respiratory function in critically ill patients [7].

Hemodynamic Stability

The hemodynamic stability of ketamine in critically ill patients is a topic of ongoing research and discussion. Generally, ketamine has a more favorable hemodynamic profile compared to other sedatives like propofol, as it is not associated with the same degree of hypotension. This makes ketamine a theoretically better option for sedation in the ICU setting [19]. However, the evidence is not entirely clear-cut. A retrospective study found a trend toward increased vasopressor requirements in patients receiving continuous ketamine infusion compared to those receiving propofol and midazolam, although the difference was not statistically significant. The authors note that this finding could be due to selection bias and the study's small sample size [19]. In contrast, another study found that patients who received ketamine for emergency intubation experienced post-induction hypotension and cardiovascular collapse significantly more often than those who received etomidate. The authors hypothesize that this may be due to ketamine's direct negative inotropic effects, which can predominate in critically ill patients with an inadequate sympathetic response [20]. On the other hand, a retrospective review found ketamine to be efficacious for continuous sedation in mechanically ventilated patients, with a favorable safety profile and no significant hemodynamic effects. This suggests that ketamine may be a viable option for sedation in the ICU, particularly in patients with cardiovascular instability [21].

Potential Neuroprotective Effects

Ketamine exhibits both neuroprotective and neurotoxic properties, depending on the dose and context. Its neuroprotective mechanisms involve anti-inflammatory and antioxidant effects and the activation of neurotrophic signaling cascades. These neuroprotective actions are not always dependent on the NMDA receptor [22]. Ketamine has been demonstrated to prevent cognitive impairment induced by isoflurane anesthesia in rats through its anti-inflammatory, anti-apoptotic, and antioxidant effects. Additionally, ketamine can prevent cognitive impairment and neurodegeneration associated with anesthesia in developing brains by inhibiting inflammation-mediated toxicity and promoting synaptic plasticity by releasing brain-derived neurotrophic factor (BDNF) [23]. The clinical relevance of ketamine's neuroprotective properties is particularly notable in the pediatric setting, where neurotoxicity is a concern during anesthesia. Ketamine's ability to promote neuroprotection in developing brains makes it a promising candidate for pediatric anesthesia. Furthermore, ketamine's neuroprotective properties could potentially be harnessed to treat neurodegenerative diseases, such as Alzheimer's disease, by reducing inflammation and promoting neurotrophic signaling [24].

Challenges and considerations

Adverse Effects and Management Strategies

The use of ketamine as a sedative in critically ill patients presents several challenges and considerations. While ketamine has shown promise in reducing sedative requirements and improving patient outcomes, the evidence base for its use in this setting is limited. Randomized controlled trials (RCTs) are necessary to establish its safety and efficacy fully. Ketamine can cause adverse events such as tachydysrhythmias, agitation, and hallucinations, which are dose-dependent, requiring careful titration to minimize risks. The impact of ketamine on patient-centered outcomes, such as the duration of mechanical ventilation and ventilator-free days, is unclear, and more studies are needed to determine its effect on these outcomes [7]. Dosing and administration of ketamine require careful consideration. Continuous infusions of 1-2 μg/kg/min for 48 hours have been studied, but more research is needed to refine dosing regimens. Ketamine's effectiveness compared to other sedatives, such as propofol and benzodiazepines, needs further investigation. While ketamine appears to have a similar frequency of adverse events, its comparative efficacy and cost-effectiveness are still being evaluated. Ketamine should be part of a multimodal analgesic and sedative approach, as it can reduce opioid and sedative requirements, helping mitigate the risks associated with prolonged use of these agents [25]. The application of ketamine in different critical care settings, such as sepsis, neurocritical care, and post-cardiac surgery care, requires further study. High-quality clinical trials should guide its use in these settings. Addressing these challenges will help in optimizing the use of ketamine as a sedative in critically ill patients, ensuring better patient outcomes and safer care. Key adverse effects and management strategies for the use of ketamine sedation in critically ill patients include cardiovascular effects, respiratory depression, neuropsychiatric effects, and gastrointestinal effects. Management strategies involve dose titration, multimodal analgesia and sedation, monitoring and supportive care, patient selection, and an interdisciplinary approach [26].

Patient Selection Criteria

When considering the use of ketamine sedation in critically ill patients, careful candidate selection is crucial. Patients with severe comorbidities may be better managed in an inpatient setting rather than an ambulatory or office-based setting for ketamine administration. Additionally, patients with moderate to severe hepatic dysfunction, such as cirrhosis, should be excluded from receiving ketamine infusions in the outpatient setting [7]. The treatment team must have the capability to provide advanced airway management and immediate treatment for potential adverse effects, including hypoxia, apnea, hypotension, dysphoria, and dysrhythmia. Appropriate monitoring equipment, such as capnography and pulse oximetry, must be readily available. The physician or certified registered nurse anesthetist (CRNA) administering ketamine must demonstrate competency in understanding its pharmacology, proper dosing, patient selection, and patient monitoring [27]. Recent studies have highlighted the efficacy of ketamine for sedation in the prehospital setting and as a rescue medication in the emergency department for agitation and delirium. Ketamine may be faster at controlling agitation compared to standard medications. Additionally, ketamine infusions may reduce the incidence and duration of delirium in mechanically ventilated ICU patients without affecting mortality or length of stay. It can also serve as an adjunctive strategy to reduce opioid and propofol use [28].

Comparison With Other Sedative Agents

Ketamine, propofol, and benzodiazepines are commonly used sedative agents, each with distinct mechanisms of action, advantages, and drawbacks. Ketamine is a dissociative anesthetic that blocks pain signal transmission between the thalamus and cortex, leading to dissociative anesthesia and amnesia. Its key advantages include providing excellent analgesia and amnesia, which is beneficial for procedures requiring pain control and memory loss. Additionally, ketamine preserves muscle tone and protective airway reflexes, making it suitable for patients with cardiovascular instability. However, adverse effects such as tachydysrhythmias, agitation, and hallucinations can occur, particularly at higher doses [29]. Propofol is a non-barbiturate sedative-hypnotic that acts on the GABA receptor to induce sedation and anesthesia. Known for its rapid onset and short recovery time, propofol is well-suited for brief procedures. It also has antiemetic and anticonvulsant properties, reducing the risk of nausea and seizures. However, it can cause significant respiratory depression and hypotension, especially at higher doses [30]. Benzodiazepines also act on the GABA receptor, providing sedation and anxiolysis. They are effective for reducing anxiety and stress and offer smooth sedation, commonly used for preoperative sedation. Like propofol, benzodiazepines can cause respiratory depression and hypotension, particularly at higher doses [31]. When comparing these agents, ketamine is often preferred for its hemodynamic stability and analgesic properties, making it suitable for patients with cardiovascular instability and those needing pain control. Propofol is favored for its quick onset and short recovery time, which makes it ideal for rapid recovery procedures. Benzodiazepines are valued for their anxiolytic effects and smooth sedation, though they carry risks of respiratory depression and hypotension [32]. The combination of ketamine and propofol, known as "ketofol," aims to balance their adverse effects, such as propofol's respiratory depression and ketamine's sympathomimetic effects. This combination theoretically reduces adverse respiratory events and improves hemodynamic stability, but the complex dosing required for ketofol can pose challenges in clinical practice [32].

Current evidence and clinical studies

Review of Clinical Trials and Studies Evaluating Ketamine Sedation

Indications and dosing: Ketamine is widely utilized for analgesia, sedation, and analgosedation in critically ill patients, especially within surgical, trauma, and medical ICUs. The typical starting dose for continuous infusion is approximately 0.2 mg/kg/hr, with a median duration of 1.6 days. Dosing usually ranges from 0.05 mg/kg/hr to 0.4 mg/kg/hr, although some studies have explored higher doses up to 2 mg/kg/hr [33].

Impact on sedation, analgesia, and delirium: Ketamine has been shown to reduce the incidence and duration of delirium in mechanically ventilated ICU patients compared to placebo or standard sedation approaches. It may also lower the need for opioids and other sedatives, though evidence on this is mixed. As a potent analgesic, ketamine is effective both as an adjunct for pain management and as an anesthetic agent [7].

Safety and adverse effects: The frequency of adverse effects associated with continuous ketamine infusion is similar to those observed with other common sedatives like propofol and benzodiazepines. Potential side effects include tachydysrhythmias, agitation, hypersalivation, emergence reactions, laryngospasm, and vomiting. Despite these possible adverse effects, ketamine generally maintains a favorable safety profile with minimal impact on hemodynamics and agitation, and serious adverse events are rare [21]. Clinical studies and observational studies have indicated that ketamine is effective for continuous sedation in mechanically ventilated patients, with a safety profile and frequency of adverse events comparable to other sedatives. RCTs have confirmed ketamine's efficacy in achieving moderate sedation, particularly in patients who are difficult to sedate [21].

Clinical practice guidelines: In the emergency department setting, ketamine is recognized as a safe and effective option for procedural sedation in children, particularly for short, painful procedures that require immobilization. The initial dose typically ranges from 1 mg/kg to 1.5 mg/kg, with additional incremental doses as needed, up to a maximum of 4.5 mg/kg [34].

Outcomes Related to Sedation Quality, Patient Comfort, and Safety

Outcomes related to sedation quality, patient comfort, and safety in critically ill patients are crucial for optimizing care and improving patient outcomes. One key finding is that deeper sedation levels are associated with higher mortality than lighter sedation levels, suggesting that maintaining lighter sedation depths may benefit patient recovery and survival. Additionally, agitation is linked to higher mortality in critically ill patients, underscoring the importance of managing patient comfort and reducing agitation [35]. The choice of sedative medications also impacts patient outcomes. For instance, the use of benzodiazepines, even at higher cumulative doses, is associated with a 41% higher mortality compared to lower doses. Conversely, the use of haloperidol is associated with a 70% lower mortality rate. This highlights the need for careful consideration of sedative medication choices and dosing. Furthermore, cumulative doses of benzodiazepines and opioids are typically high in resource-limited settings, approximately doubling by day 28. This emphasizes the importance of optimizing sedation practices and minimizing the use of these medications [36]. Sedation monitoring and management are critical for achieving optimal sedation depth and improving patient outcomes. Protocolized sedation assessment and monitoring using validated scales, such as the Richmond Agitation-Sedation Scale (RASS) and the Sedation-Agitation Scale, are recommended to target the optimal sedation depth. Sedation with daily interruption achieves lighter sedation and enables patient awakening and neurological assessment. Optimizing sedation practices and implementing delirium screening are associated with improved outcomes, including lower incidence of delirium, fewer days on mechanical ventilation, and reduced mortality. These findings emphasize the importance of structured sedation monitoring and management to enhance patient comfort, safety, and survival in critically ill patients [35].

Future directions and research needs

Emerging Trends in Ketamine Use in Critical Care

Emerging trends in ketamine use in critical care highlight its growing role as an adjunct for analgosedation, its expanding applications in procedural sedation, and its potential to reduce delirium and opioid use. Ketamine's unique pharmacological properties, including its rapid sedative, analgesic, and amnesic effects and its ability to induce bronchodilation and maintain sympathetic nervous system tone, make it a versatile drug in critical care settings [7]. Recent studies have uncovered previously unrecognized neuroprotective and anti-inflammatory properties of ketamine, which are beneficial in critical care scenarios. Ketamine is utilized for sedation, analgesia, and treating persistent bronchospasm. It has also demonstrated efficacy with propofol (ketofol) for short-term sedation in critical care populations [3]. Ketamine is recommended as a non-benzodiazepine sedative for managing pain, agitation, and delirium in critically ill patients. Although not routinely recommended for delirium treatment, ketamine shows promise in managing agitated patients. Studies indicate that ketamine can reduce the incidence and duration of delirium without impacting mortality rates or length of stay in the ICU [7]. Ketamine's role as an adjunct to reduce opioid and propofol use in surgical or trauma-intubated patients is well-documented. It is also effective in procedural sedation, offering faster control of agitation compared to standard emergency department medications. A meta-analysis found that intramuscular administration of ketamine achieved adequate sedation in a mean time of seven minutes, which is faster than traditional antipsychotics and benzodiazepines [7]. A multicenter retrospective review of ketamine use in critically ill patients detailed its indications, dosing, duration, adverse effects, patient outcomes, and time spent within target pain/sedation score ranges. The study found that ketamine was primarily used for analgesia and sedation, associated with increased time spent in goal pain/sedation score ranges and decreased cumulative exposure to other analgesic and sedative agents [33].

Areas for Further Research and Clinical Trials

The current research on ketamine use for sedation in critically ill patients identifies several areas that warrant further investigation through future research and clinical trials. Larger, high-quality RCTs are needed to directly compare ketamine's efficacy and safety with standard sedatives like propofol and benzodiazepines in critically ill populations. These trials should focus on patient-centered outcomes such as duration of mechanical ventilation, incidence of delirium, ICU length of stay, and mortality [37]. Further research is required to determine optimal dosing regimens and titration protocols for continuous ketamine infusions. This includes assessing appropriate starting doses, dose ranges, and maximum doses to balance efficacy and safety. Additionally, the role of ketamine sedation should be explored in specific patient populations, such as those with traumatic brain injury or other neurological conditions, where its potential neuroprotective effects could be particularly beneficial. Pediatric studies are currently lacking, and more research is needed to evaluate the safety and efficacy of ketamine sedation in critically ill children [38]. Ongoing research into the neurological mechanisms underlying ketamine's sedative, analgesic, and delirium-reducing effects in critically ill patients is also warranted. Identifying relevant biomarkers could help predict and optimize individual patient responses. While the short-term safety of ketamine appears comparable to other sedatives, its long-term effects on the brain and other organ systems, particularly concerning the potential for abuse and misuse, require further study [7].

Potential Advancements in Sedation Protocols

Potential advancements in sedation protocols focus on developing new sedatives and analgesics and improving monitoring and administration practices. Remimazolam, a short-acting benzodiazepine, may offer enhanced sedation with fewer side effects compared to midazolam. Ciprofol, another short-acting anesthetic agent, is also being investigated for its efficacy in procedural sedation. Additionally, oliceridine, a novel analgesic, has the potential to broaden current practices in procedural sedation [39]. The use of capnography for monitoring represents another significant advancement. Capnography is becoming a core monitoring technique for sedation, providing real-time monitoring of carbon dioxide levels to ensure patient safety [40]. Another promising development is the non-anesthetist administration of propofol (NAAP). This approach allows nurses and other non-anesthetist personnel to administer propofol, potentially making sedation more efficient and safer compared to traditional anesthetist-administered sedation. Standardization and governance of sedation services are also crucial advancements. Establishing sedation committees and governance structures can oversee sedation processes, ensuring continuous quality improvement and patient safety [41]. Integrating multidisciplinary teams, including sedation practitioners, anesthetists, medical staff, and nurses, is another potential advancement. These teams can collaboratively manage procedural sedation and analgesia in emergency settings. Finally, developing local and national guidelines for procedural sedation is an important advancement. Tailored to the specific needs and resources of different healthcare settings, these guidelines can improve patient safety and enhance the overall quality of sedation practices [42].

## Conclusions

In conclusion, ketamine presents itself as a valuable sedative option in the management of critically ill patients, offering distinct advantages over traditional agents in terms of maintaining respiratory and cardiovascular stability. Its unique pharmacological profile, characterized by dissociative anesthesia and analgesic properties, makes it a versatile tool in the ICU setting, where balancing sedation depth with patient safety is paramount. While challenges such as potential psychomimetic effects and variability in patient response exist, ongoing research and clinical experience continue to refine our understanding and utilization of ketamine in critical care. Further investigation into optimal dosing strategies, patient selection criteria, and long-term outcomes will be essential to fully harnessing ketamine's potential and integrating it effectively into sedation protocols for critically ill patients. By doing so, we can improve the immediate management and long-term prognosis of patients requiring intensive care sedation.
